# Multi‐Institutional Phase II Study on the Efficacy and Safety of Dynamic Tumor‐Tracking, Moderately Hypofractionated Intensity‐Modulated Radiotherapy in Patients With Locally Advanced Pancreatic Cancer

**DOI:** 10.1002/cam4.70648

**Published:** 2025-02-05

**Authors:** Michio Yoshimura, Masahiro Hiraoka, Masaki Kokubo, Takashi Sakamoto, Katsuyuki Karasawa, Yukinori Matsuo, Mitsuhiro Nakamura, Nobutaka Mukumoto, Satoshi Morita, Takashi Mizowaki

**Affiliations:** ^1^ Department of Radiation Oncology and Image‐Applied Therapy, Graduate School of Medicine Kyoto University Kyoto Japan; ^2^ Department of Radiation Oncology Kobe City Medical Center General Hospital Kobe Japan; ^3^ Department of Radiation Oncology Kyoto Katsura Hospital Kyoto Japan; ^4^ Division of Radiation Oncology, Department of Radiology Tokyo Metropolitan Cancer and Infectious Diseases Center Komagome Hospital Tokyo Japan; ^5^ Department of Advanced Medical Physics, Graduate School of Medicine Kyoto University Kyoto Japan; ^6^ Department of Biomedical Statistics and Bioinformatics, Graduate School of Medicine Kyoto University Kyoto Japan

**Keywords:** dynamic tumor tracking, intensity‐modulated radiotherapy, pancreatic cancer, respiratory motion management

## Abstract

**Background:**

For delivering high radiation doses to pancreatic tumors, organ motion management is indispensable; however, studies on this are limited. We aimed to evaluate the efficacy and safety of dynamic tumor tracking (DTT) moderately hypofractionated intensity‐modulated radiotherapy (IMRT) in patients with locally advanced pancreatic cancer (LAPC).

**Methods:**

Patients with histological confirmation for LAPC were included. A linac system, which was mounted with a gimbal function, was used for DTT‐IMRT. The prescribed dose was 48 Gy in 15 fractions. The primary endpoint was the 1‐year rate of freedom from locoregional progression (FFLP).

**Results:**

DTT‐IMRT was successfully administered in 25 patients enrolled from four institutions. The median range of respiratory motion during DTT‐IMRT was 9.8 mm (range: 3.5–27.3 mm), and the median tracking accuracy was 2.6 mm (range: 0.7–5.2 mm). With a median follow‐up period of 13.9 months, the 1‐year FFLP rate was 75.3% (lower limit of one‐sided 80% confidence interval [CI]: 60.2%), which satisfied the predetermined primary endpoint. One‐year locoregional progression‐free survival, progression‐free survival, and overall survival were 56.0% (95% CI: 34.8%–72.7%), 44.0% (95% CI: 24.5%–61.9%), and 60.0% (95% CI: 38.4%–76.1%), respectively. Regarding nonhematologic toxicities, grade 3 acute gastrointestinal (GI) toxicity was observed in two patients (8%), and two patients (8%) each experienced grade 3 late GI and non‐GI toxicities. No grade 4 or 5 nonhematologic toxicities were observed.

**Conclusions:**

DTT moderately hypofractionated IMRT shows preferable locoregional control without significant toxicity in patients with LAPC.

**Trial Registration:**

UMIN000017521

## 1 Introduction

Pancreatic cancer is a solid organ cancer with the poorest prognosis worldwide [1]. According to the National Comprehensive Cancer Network (NCCN) guidelines, its treatment strategy depends on the resectability status of the tumor [2]. Surgery is the best cure for pancreatic cancer when distant metastases are not present. However, chemotherapy and/or radiotherapy are the treatment options for unresectable locally advanced pancreatic cancer (LAPC), wherein the tumor is in contact with main arteries such as the superior mesenteric artery (SMA) or celiac artery (CA) at an angle > 180° [2].

However, LAPC treated with chemoradiotherapy using the conventional dose fractionation (50–54 Gy in 25–30 fractions) has a poor prognosis with a median survival of 8.2–17.6 months [3, 4, 5, 6]. Although Krishnan et al. [7] reported that radiation dose escalation could improve overall survival (OS) and locoregional progression‐free survival (LPFS) in patients with LAPC, high‐precision techniques, including intensity‐modulated radiotherapy (IMRT), should be used for dose‐escalated irradiation. The efficacy of IMRT in patients with pancreatic cancer has been demonstrated in several studies [8]. Moreover, the Radiation Therapy Oncology Group (RTOG) 97‐04 trial showed that IMRT decreased the acute gastrointestinal (GI) toxicity rate in patients with pancreatic cancer compared to that after conventional three‐dimensional conformal radiation therapy [9]. Additionally, we have previously reported that dose‐escalated moderately hypofractionated IMRT could improve the survival of patients with LAPC showing acceptable toxicities [9, 10].

Organ motion management is indispensable for delivering high‐dose irradiation to pancreatic tumors for several reasons. First, the pancreas is surrounded by critical organs, such as the stomach, duodenum, liver, kidney, and spinal cord, all of which are radiosensitive. Second, both the pancreas and surrounding organs have intrafractional motion due to respiratory and peristaltic movements and interfractional motion resulting from changes in the filling contents of the GI organs. Expansion of internal target volume (ITV) margins is a conventional method of organ motion management; however, it is unfavorable due to side effects in normal tissues. Other options include abdominal compression, breath‐hold, respiratory gating, and dynamic tumor tracking (DTT) techniques [10, 11, 12]. Among these techniques, DTT has been proven to be superior in terms of daily treatment time and compliance with patients' respiratory control [10]. Moreover, DTT can reduce the ITV margin while maintaining dose delivery to the target [10, 13, 14]. However, since several complicated procedures and strict quality assurance/quality control (QA/QC) standards are required to perform DTT‐IMRT, only a few studies on DTT‐IMRT for pancreatic cancer have been reported to date [11, 15].

To the best of our knowledge, no prospective DTT‐IMRT study on patients with pancreatic cancer in a multi‐institutional setting has been reported thus far. Therefore, we conducted a phase II study to evaluate the safety and efficacy of DTT‐IMRT in patients with LAPC.

## 2 Methods

### 2.1 Patients

The eligibility criteria for patients with LAPC were: Patients with clinical T3‐4N0‐1 (stage II or III) LAPC (UICC 7th version); histological confirmation of the tumor as adenocarcinoma or adenosquamous carcinoma; the tumor was located in the pancreatic head, body, or tail with no distant metastases on the contrast‐enhanced dynamic computed tomography (CT), magnetic resonance imaging (MRI), and upper gastrointestinal endoscopy; SMA or CA encasement > 180° or determined as an inoperable disease by a multidisciplinary panel; disease extension ≤ 8 cm; age ≥ 20 < 80 years; ECOG performance status of 0 or 1; no prior surgery or radiotherapy for pancreatic cancer; no prior chemotherapy was desirable, but one course of gemcitabine, tegafur/gimeracil/oteracil potassium (S‐1), or gemcitabine/nab‐paclitaxel was acceptable; expected range of respiratory motion ≥ 10 mm; and confirmation that DTT‐IMRT was realizable after the insertion of gold fiducial marker.

The exclusion criteria were as follows: Patients with pulmonary fibrosis or interstitial pneumonia; prior history of radiotherapy to the upper abdomen; severe comorbidities such as diabetes mellitus, collagen disease, heart, and renal failure; moderate or higher ascites or pleural effusion; tumor invasion to gastrointestinal mucosa and other inappropriate factors for the study.

This study was conducted in accordance with the tenets of the Declaration of Helsinki. The Ethics Committees of the participating institutions approved the study protocol. All patients provided written informed consent before participation. The study was registered in the UMIN Clinical Trials Registry (UMIN000017521).

#### 2.1.1 Study Protocol

**FIGURE 1:**
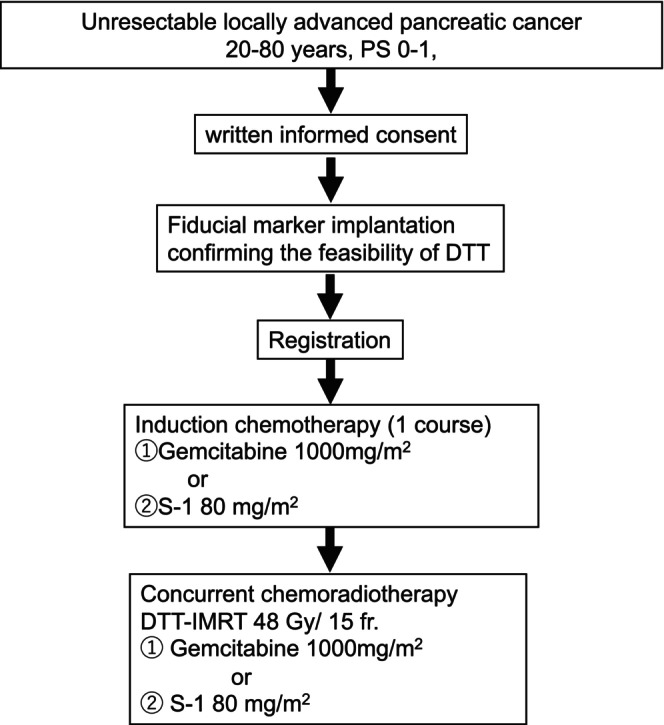
Schema of the protocol treatment.

After consent acquisition, a gold marker (0.5 or 0.75 × 10 mm, Visicoil, IBA dosimetry, GmbH, Germany) was implanted endoscopically or percutaneously inside or near the primary tumor as an internal surrogate marker for determining the position of the primary tumor. After confirming dynamic tumor tracking (DTT), this study enrolled the patients. After one course of administering induction chemotherapy with gemcitabine or S‐1, DTT‐IMRT (48 Gy in 15 fractions) was delivered concurrently with weekly gemcitabine or S‐1. The schema of the treatment protocol used in this study is shown in Figure 1.

#### 2.1.2 Treatment Planning

**FIGURE 2:**
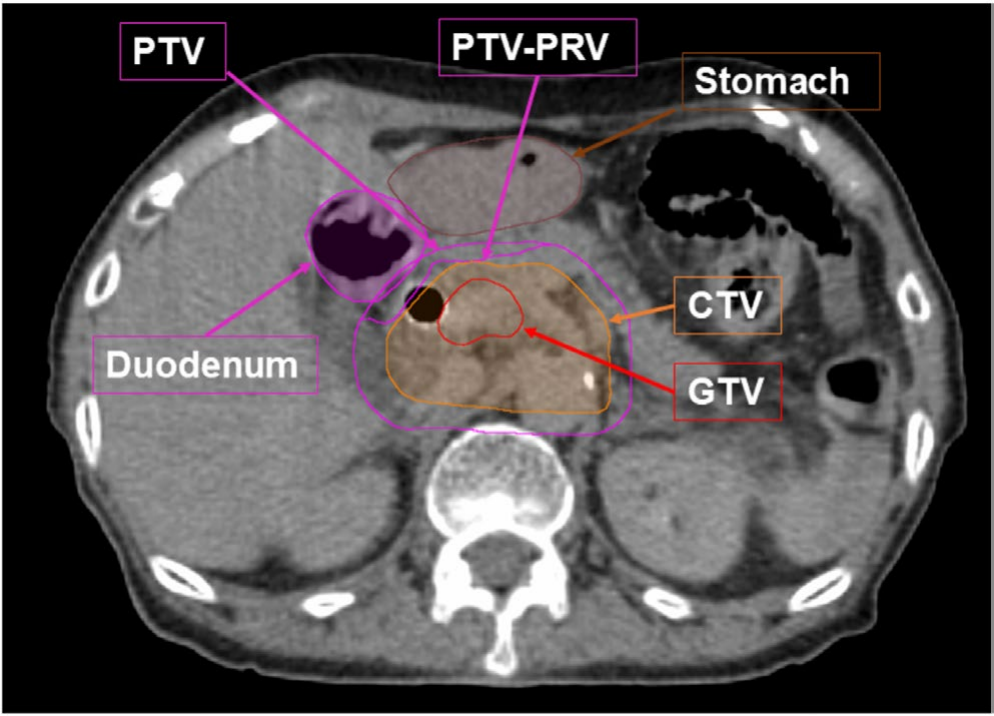
Representative structure contouring. PTV‐PRV: The volume which subtracted (stomach+5 mm) and (duodenum+3 mm) from PTV.

We previously reported the planning procedures for DTT‐IMRT [11, 15]. Briefly, after a minimum 3 h of fasting, patients underwent examination using contrast‐enhanced CT with end‐expiratory breath‐holding and subsequent free‐breathing four‐dimensional CT (4D‐CT). The periodic whole images were sorted into 10 phased bins of 4D‐CT images. The gross tumor volume (GTV) was defined as the primary pancreatic tumor and metastatic lymph nodes. The clinical target volume (CTV) included the GTV + a margin of 5 mm in all directions, the retropancreatic area, and the para‐aortic lymph node area between the CA and SMA. We contoured GTV, CTV, and the upper GI organs at risk (OARs) on all 10 respiratory phases of 4D‐CT and overlaid them onto the mid‐ventilation phase image. Considering the patients' setup error, 4D modeling error, mechanical error, and baseline drift of the abdominal position, 5 mm of the planning target volume (PTV) margin was added to the CTV. PTV‐PRV (planning organ at risk volume) was defined as the volume subtracted from the PTV of the stomach plus 5 mm and duodenum plus 3 mm margins (Figure 2). IMRT planning was conducted with iPlan RT Dose (Brainlab AG, Munich, Germany), utilizing the X‐ray Voxel Monte Carlo (XVMC) algorithm for radiation dose calculation. The spatial resolution and deviation were ≤ 2 mm and ≤ 2%, respectively. For the prescription dose, we defined that D95% (irradiation dose that included 95% volume of the target) for PTV‐PRV equals 48 Gy, and D98% for PTV was 36 Gy or more with the simultaneously integrated boost IMRT technique. The dose constraints of the PTVs and OARs are listed in Table 1.

**TABLE 1:** Dose constraints and reported values for PTV, OAR, and PRV and volumes of GTV, CTV, and PTV (*n* = 25).

Structure	Constraints	Mean	Range
PTV			
D98%	≥ 36 Gy [≥ 34.2 Gy]	36.0	34.2–38.7
PTV‐PRV			
D95%	= 48 Gy [≥ 45.6 Gy]	47.4	45.6–48.1
D50%	≒ 51 Gy	50.6	48.1–52.4
Stomach			
V39 Gy	≤ 25 cc	1.2	0.0–7.9
V42 Gy	≤ 5 cc	0.2	0.0–1.7
V45 Gy	≤ 1 cc	0.0	0.0–0.4
Duodenum			
V39 Gy	≤ 25 cc	4.0	0.0–15.2
V42 Gy	≤ 5 cc	0.9	0.0–4.1
V45 Gy	≤ 1 cc	0.1	0.0–0.8
Stomach (PRV)			
V36 Gy	≤ 45 cc	28.3	0.0–44.6
V39 Gy	≤ 30 cc	14.4	0.0–27.0
Duodenum (PRV)			
V36 Gy	≤ 45 cc	28.9	6.2–44.8
V39 Gy	≤ 30 cc	15.4	2.8–24.0
Spinal cord			
Dmax	< 36 Gy	31.9	20.2–35.5
Spinal cord (PRV)			
D2cc	< 39 Gy	29.3	17.4–34.2
Liver			
Dmean	≤ 30 Gy	9.1	1.2–17.3
Ipsilateral kidney			
V20 Gy	≤ 30%	19.6	3.0–26.9

Volumes	Mean	SD	Min	Max
GTV (cm^3^)	44.3	32.1	7.7	133.8
CTV (cm^3^)	127.0	53.2	62.3	275.9
PTV (cm^3^)	241.7	79.5	137.6	482.3

*Note:* [ ]: Variation acceptable.

Abbreviations: CTV = clinical target volume; *D*max = the maximum dose to the volume; *D*mean = the mean dose to the volume; Dx% = dose covering x% of the volume; D2cc = the dose covering the ≥ 2 cc of the volume; GTV = gross tumor volume; OAR = organ at risk; PRV = planning organ at risk volume; PTV = planning target volume; VxGy = volume covered by the x‐Gy isodose.

#### 2.1.3 DTT‐IMRT Delivery

**FIGURE 3:**
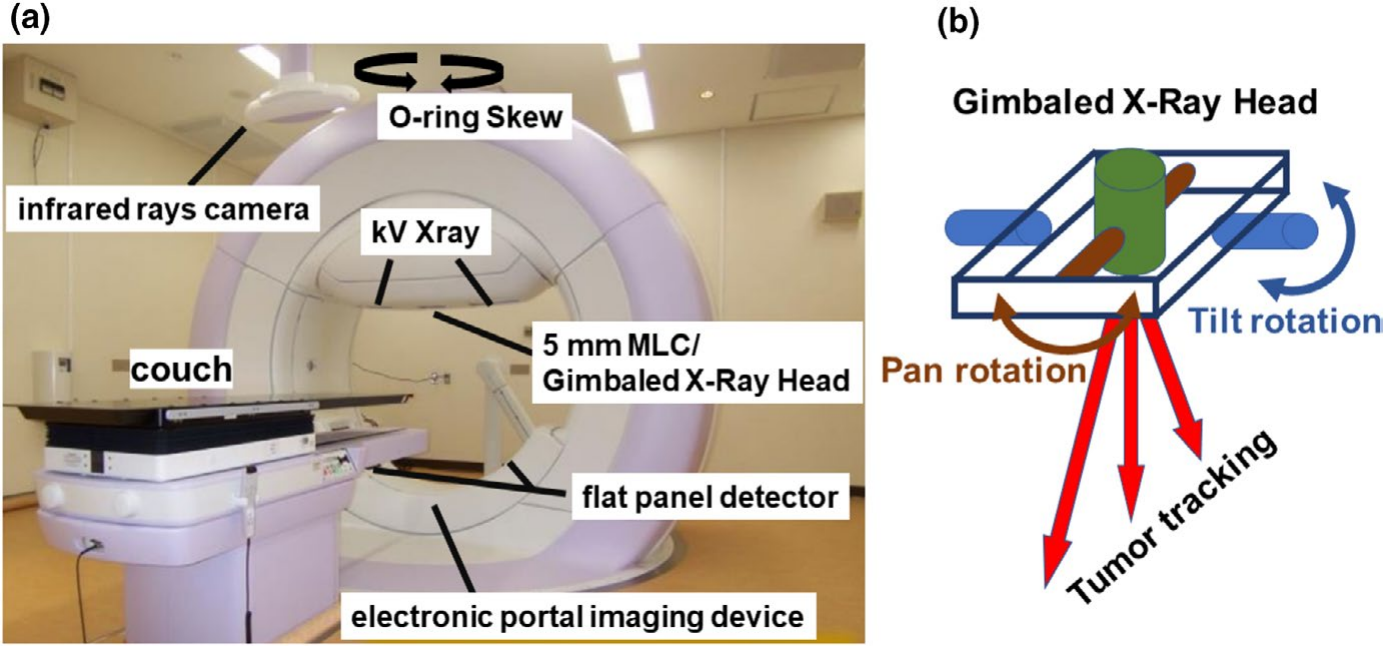
(a) Exterior view and basic structure of Vero4DRT. (b) Pan and tilt rotations of the gimbaled X‐ray head of Vero4DRT.

The DTT‐IMRT delivery methods have been elaborated in our previous reports [11, 15]. Briefly, patients were immobilized using BodyFix, and initial setup errors were corrected based on bony structures. A 4D model was constructed before daily irradiation. Orthogonal X‐ray images mounted with the gantry and an infrared (IR) marker on the upper abdomen were utilized to establish a correlation 4D model between the internal gold fiducial marker and the abdominal wall. The future target position was predicted using a pre‐established correlation model reliant on the movement of the IR marker. The gimbaled gantry head of Vero4DRT (Hitachi Ltd., Tokyo, Japan) can precisely target moving structures during free breathing by utilizing a predictive correlation 4D model based on the infrared (IR) marker positioned on the upper abdomen (Figure 3a,b). Throughout beam delivery, we continuously monitored the position of the fiducial marker using kV and MV X‐ray images, updating the predicted marker position overlaid on the kV images using a 3 mm radius circle. If the gold fiducial marker deviated frequently from this circle, irradiation was paused, and the correlation 4D model was reconstructed.

#### 2.1.4 Chemotherapeutic Regimen

For induction chemotherapy, one course of gemcitabine (1000 mg/m^2^) was administered intravenously once a week thrice, followed by 1 week of rest, or S‐1 (80 mg/m^2^/day) was administered orally twice daily for 2 weeks, followed by 1 week of rest. During radiotherapy, weekly intravenous gemcitabine (1000 mg/m^2^) or oral S‐1 (80 mg/m^2^/day) twice daily on weekdays was administered concurrently. After the termination of chemoradiotherapy, maintenance chemotherapy, such as gemcitabine with nab‐paclitaxel, gemcitabine alone, or S‐1, was repeated as long as possible.

#### 2.1.5 QA Program

We commissioned all four facilities to undergo an IMRT dose‐delivery test conducted by Imaging and Radiation Oncology Core in Houston, USA. Before the commencement of this study, all facilities successfully completed the examination using their respective systems, including Vero4DRT and iPlan RT Dose, utilizing the XVMC algorithm. As part of the test protocol, facilities were required to provide dose–volume indices for both PTVs and OARs and log files detailing the accuracy of tumor tracking during DTT‐IMRT delivery. Target positions were determined from stereo‐fluoroscopic images captured every second, and tracked positions were obtained from the radiation treatment beams using the gimbal system, with data extracted from the log files. We defined the respiratory motion range as the 95th percentile of the target position during treatment and tracking accuracy as the 95th percentile difference between detected and tracked target positions.

#### 2.1.6 Follow‐Up

After registration of the first five cases, a safety assessment was performed for 90 days. Registration was reinitiated after the safety confirmation of all five patients. Contrast‐enhanced dynamic CT and blood investigations for tumor markers were performed at least once every 3 months. Locoregional tumor progression and distant metastases were determined based on CT or fluorodeoxyglucose‐positron emission tomography (FDG‐PET) findings.

#### 2.1.7 Endpoints

The primary endpoint was the 1 year rate of freedom from locoregional progression (FFLP). The required sample size was estimated based on an expected value of 80%, a threshold value of 60%, one‐sided alpha of 0.10, and 80% power. Therefore, the number of enrolled patients was set to 25, in case one patient was to drop out from the analysis. Secondary endpoints included LPFS, PFS, OS, serious acute and late adverse events, treatment‐related death, and the accuracy of tumor tracking. Adverse events were graded according to the Common Terminology Criteria for Adverse Events (version 4.0).

#### 2.1.8 Statistical Analysis

FFLP was defined as the time from initiation of induction chemotherapy until locoregional progression and was censored at the last follow‐up or date of death. Locoregional progression was defined as progressive disease of the primary tumor, retropancreatic area, or para‐aortic lymph node areas between CA and SMA.

OS was calculated from the starting date of induction chemotherapy to the date of death due to any cause and was censored at the last follow‐up visit for living patients. LPFS and PFS were calculated from the starting date of induction chemotherapy to locoregional disease progression or death and disease progression or death, respectively. Disease progression was defined as the evidence of disease progression on CT or FDG‐PET using the Response Evaluation Criteria in Solid Tumors [16]. Statistical analyses were performed using EZR version 1.60 (Saitama Medical Center, Jichi Medical University, Saitama, Japan). The Kaplan–Meier technique was used to estimate FFLP, LPFS, PFS, and OS.

## 3 Results

In total, 25 eligible patients were enrolled from four different facilities between October 2015 and December 2018: 13 male and 12 female. The study registration process took longer than we had initially planned because many candidates did not meet the eligibility criteria of the expected range of respiratory motion ≥ 10 mm or receiving no more than one course of induction chemotherapy at the time of the first referral. The median age of the study population was 69 years (range: 52–77 years), and all patients were histologically diagnosed with adenocarcinoma. The primary tumor was in the pancreatic head and body in 14 and 11 patients, respectively. The median length of the major axis of the primary tumor was 33 mm (range: 20–70 mm). According to the 7th edition of the Union for International Cancer Control (UICC), 3, 1, and 21 cases were classified as clinical stages IIA, IIB, and III, respectively (Table 2).

**TABLE 2:** Patient, tumor, and treatment characteristics (*n* = 25).

Gender	Male/female	13/12
Age (years)	Median (range)	69 (52–77)
Tumor location	Head/body	14/11
Tumor size (major axis) (mm)	Median (range)	33 (20–70)
T stage	3/4	4/21
N stage	0/1	19/6
Clinical stage	IIA/IIB/III	3/1/21
Induction chemotherapy	Gemcitabine/S‐1	19/6
Concurrent chemotherapy	Gemcitabine/S‐1	18/7

*Note:* TNM stage is defined in UICC 7th edition.

**FIGURE 4:**
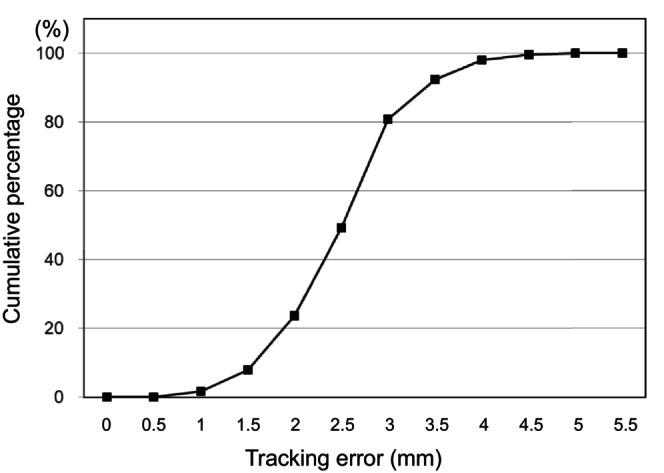
Accumulated probability histogram as a function of tracking error.

The goals for dose–volume indices were met for all 25 patients. Mean (range) values for GTV, CTV, and PTV were 44.3 cm^3^ (7.7–134 cm^3^), 127 cm^3^ (62.3–276 cm^3^), and 242 cm^3^ (138–482 cm^3^), respectively (Table 1). DTT‐IMRT was successfully performed in all patients. The median range of respiratory motion during DTT‐IMRT was 9.8 mm (range: 3.5–27.3 mm). The median tracking accuracy was 2.6 mm (range: 0.7–5.2 mm). Of the 375 fractions (15 fractions × 25 patients), the tracking error was ≤ 3 mm and ≤ 4 mm in 80.8% and 96.5% of the fractions, respectively (Figure 4).

**FIGURE 5:**
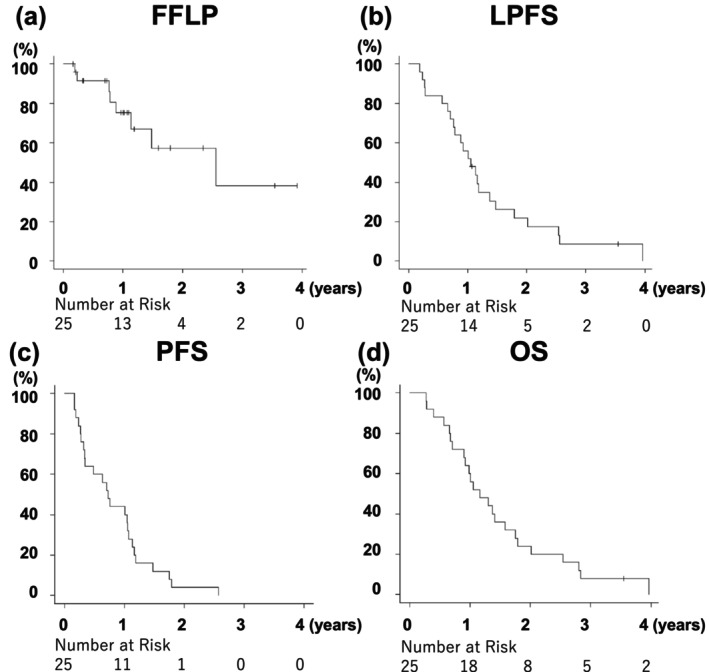
Freedom from locoregional progression (FFLP) (a), locoregional progression‐free survival (LPFS) (b), progression‐free survival (PFS) (c), and overall survival (OS) (d) from the initiation of induction chemotherapy.

The median follow‐up period was 13.9 months (range: 3.3–47.5 months). The FFLP rate at 1 year was 75.3% (lower limit of the one‐sided 90% confidence interval [CI]: 60.2%; Figure 5a). One‐year LPFS, PFS, and OS were 56.0% (95% CI: 34.8%–72.7%), 44.0% (95% CI: 24.5%–61.9%), and 60.0% (95% CI: 38.4%–76.1%), respectively. The median survival time (MST) was 13.9 months (95% CI: 10.8–21.0 months) (Figure 5a−d). Conversion surgery was performed in three patients when no disease progression was observed. Twenty‐four patients died at the data cutoff, and 22 of 25 patients had recurrence. The first recurrence site was distant metastases in 16 patients (peritoneal dissemination: Six, liver: five, lung: five, bone: one, [one duplicate]), locoregional progression in three patients, and both distant metastasis and locoregional progression in three patients.

Regarding nonhematologic toxicities, acute grade 3 GI toxicity (loss of appetite and vomiting) was observed in two patients (8%). Late grade 3 GI toxicity (gastric ulcer and duodenal ulcer) and non‐GI toxicity (biliary tract infection and ascites) were observed in two (8%) patients each. No grade 4 or 5 nonhematologic toxicities were observed (Table 3).

**TABLE 3:** Patients with grade 3 or grades 4 and 5 nonhematologic toxicity.

Nonhematologic toxicity		Grade 3	Grades 4 and 5
		No (%)	No (%)
Acute toxicity			
Nausea		1 (4%)	0
Vomiting		2 (8%)	0
Appetite loss		2 (8%)	0
Weight loss		1 (4%)	0
Biliary infection		1 (4%)	0
Ascites		1 (4%)	0
Late toxicity			
Gastric ulcer		1 (4%)	0
Duodenal ulcer		1 (4%)	0

## 4 Discussion

To the best of our knowledge, this is the first multi‐institutional clinical trial on DTT‐IMRT for LAPC.

In this study, we used moderately hypofractionated IMRT with an escalated irradiation dose. IMRT is one of the promising radiation therapy techniques for dose escalation in LAPC, along with stereotactic body radiotherapy (SBRT). However, unlike SBRT, moderately fractionated IMRT has the advantage of concurrent chemotherapy use. Several studies have reported that IMRT with concurrent chemotherapy can achieve favorable local control by reducing the frequency of GI toxicities associated with LAPC [8, 17]. We administered 48 Gy in 15 fractions, which is equivalent to 63.4 Gy at BED_10_ and almost comparable to 70 Gy at BED_10_, considering that the radiotherapy period was shortened by more than half a month to suppress tumor repopulation. Krishnan et al. [7] reported that delivering > 70 Gy at BED_10_ was ideal for improving survival after radiotherapy for LAPC. However, shortening the duration of radiation treatment with hypofractionated regimens is beneficial for patients with intractable LAPC as radiotherapy can be completed before the occurrence of adverse events, such as severe appetite loss or nausea. Moreover, intensive chemotherapy can be reinitiated at an early stage. In addition, a shorter duration of radiotherapy can prevent the impairment of a patient's quality of life. We have previously reported the preliminary favorable outcomes of moderately hypofractionated IMRT for LAPC under the condition of end exhalation breath‐hold or DTT methods, which was 92.3% of 1‐year OS rates with only 4% of late grade 3 and no grade 4 or 5 GI toxicity [18].

Moreover, DTT was used for respiratory motion management. DTT‐IMRT was successfully delivered in all patients with favorable tracking accuracy, which represented the median tracking error of 2.6 mm with ≤ 3 mm and ≤ 4 mm of tracking error in 80.8% and 96.5% of the fractions, respectively (Figure 4). Since the pancreas moves with respiration and is surrounded by radiosensitive normal organs, such as the stomach and duodenum, respiratory motion management is indispensable for dose‐escalated irradiation of LAPC. Among the several options for respiratory motion management in radiotherapy for pancreatic cancer, DTT has the advantages of shorter treatment time, no breath‐hold burden, and narrower ITV margins while maintaining dose coverage to the target [10] However, DTT provides the least evidence because fiducial markers must be implanted, and complicated procedures are required in addition to special radiotherapy equipment; furthermore, DTT requires an extremely strict QA program [19] We used a uniform QA program in this study at all attending facilities.

The FFLP rate at 1 year in this study was 75.3%, which met the predetermined primary endpoint. Previous studies showed that the 1‐year FFLP rate was 50%–85% in patients with LAPC treated with definitive chemoradiotherapy [7, 20, 21, 22]. Compared to previous reports, the FFLP rate in our study was favorable. However, the MST was 13.9 months, which was average, considering previous studies that reported an MST of 7.7–23 months for IMRT in patients with LAPC [21, 23]. As demonstrated in the LAP07 trial, an improvement in the local control rate does not lead to better OS [24]. However, whether local control translates to OS after definitive chemoradiotherapy for patients with LAPC remains unclear. Therefore, optimal induction and concurrent chemotherapy regimens along with radiotherapy should be considered. Regarding nonhematologic toxicities, our study showed that acute grade 3 GI and non‐GI toxicities were observed in 8% of the study participants each, whereas no grade 4 or 5 nonhematologic toxicities were discerned. Krishnan et al. [7] reported only 2% of acute grade 3–4 GI toxicities, whereas other studies reported that acute grade 3–4 GI toxicities occurred in 9%–25% of the patients [7, 21, 25]. In addition, late grade 3 GI toxicities were observed in 8% of the study participants, and no late grade 4 or 5 toxicities were observed. The reported rate of grade 3–4 GI toxicity of IMRT for LAPC is 2%–24%, [7, 22, 23, 25] and a representative study of dose‐escalated IMRT for LAPC showed grade 3 upper GI bleeding was observed in 8% patients [26]. The frequency of toxicities in our study was satisfactory despite dose escalation.

Nonetheless, this study had a few limitations. First, the number of patients was limited, which implies that the outcomes may depend on patient selection. The participants had potential distant metastases at the time of initial diagnosis, and 76% (19/25) patients had distant metastases at the time of the first recurrence. Second, we used monotherapy, with gemcitabine or S‐1 as induction chemotherapy. However, the NCCN guidelines currently recommend the use of FOLFIRINOX, modified FOLFIRINOX, or gemcitabine + albumin‐bound paclitaxel before chemoradiation in LAPC [2]. Moreover, the Japan Clinical Oncology Group (JCOG) reported that gemcitabine alone as induction chemotherapy is not recommended for UR‐LAPC treated with chemoradiotherapy (JCOG1106) [27], Thus, the induction chemotherapy regimens of our study are weaker than the current standard of induction chemotherapy for LAPC. As a future strategy, the intensity of induction chemotherapy should be increased in the radiation treatment of LAPC by using FOLFIRINOX, modified FOLFIRINOX, or gemcitabine + albumin‐bound paclitaxel in addition to controlling locoregional lesions. Next, this study did not incorporate the latest dose‐escalating radiation treatment strategies. In recent years, several studies attempted to further escalate the irradiation dose for LAPC and reported promising results. For example, Reyngold et al. delivered 75 Gy in 25 fractions for tumors < 1 cm away from the stomach or intestines and 67.5 Gy in 15 fractions for tumors ≥ 1 cm away using conventional C‐arm linacs, while Lee et al. and Chuong et al. escalated doses to 40 Gy or 50 Gy in five fractions using CT‐ or MR‐guided adaptive radiation therapy [26, 28, 29]. Since the treatment methods of radiation dose escalation and induction chemotherapy have changed since this study was planned, a gap has emerged between our treatment method and the current state‐of‐the‐art treatment methods. In the future, treatment strategies to integrate these newest methods with this dynamic tumor tracking technology should be developed. Finally, the development and sale of the Vero4DRT system was completed in 2016 [19]. However, 18 systems remain operational worldwide. Additionally, a novel linac with a gimbal system based on the Vero4DRT was launched in Japan in 2023 and is expected to be available worldwide soon. This new linac system introduced dynamic tumor tracking technology, and our results are expected to contribute to the development of the dynamic tumor tracking techniques of chemoradiotherapy for thoracoabdominal tumors, including LAPC in the future, which would benefit both patients with cancer and medical professions.

In conclusion, DTT moderately hypofractionated IMRT shows preferable locoregional control without significant toxicity in patients with LAPC with respiratory motion.

## Author Contributions

**Masahiro Hiraoka:** funding acquisition (equal), supervision (equal), writing – review and editing (equal). **Mitsuhiro Nakamura:** investigation (supporting), writing – review and editing (equal). **Takashi Mizowaki:** funding acquisition (equal), project administration (equal), supervision (equal), writing – review and editing (equal). **Masaki Kokubo:** resources (equal), writing – review and editing (equal). **Nobutaka Mukumoto:** investigation (supporting), writing – review and editing (equal). **Michio Yoshimura:** conceptualization (lead), investigation (lead), methodology (lead), resources (lead), software (lead), writing – original draft (lead). **Katsuyuki Karasawa:** resources (equal), writing – review and editing (equal). **Takashi Sakamoto:** resources (equal), writing – review and editing (equal). **Satoshi Morita:** formal analysis (equal), writing – review and editing (equal). **Yukinori Matsuo:** investigation (supporting), writing – review and editing (equal).

## Ethics Statement

This study was conducted in accordance with the tenets of the Declaration of Helsinki. The Ethics Committees of Kyoto University, Kobe City Medical Center General Hospital, Kyoto Katsura Hospital and Tokyo Metropolitan Cancer and Infectious Diseases Center Komagome Hospital approved the study protocol. All patients provided written informed consent before participation.

## Conflicts of Interest

Michio Yoshimura is in a speaker's bureau from Varian Medical Systems Inc.; Masaki Kokubo is in a speaker's bureau from AstraZeneca K.K. and Varian Medical Systems Inc.; Takashi Sakamoto is in a speaker's bureau from SCETI K.K., AstraZeneca K.K., Elekta K.K., Hitachi Ltd.; Mitsuhiro Nakamura receives research funding from Varian Medical Systems Inc. and a scholarship donation from Hitachi Ltd.; Yukinori Matsuo receives research funding from Varian Medical Systems Inc.; Takashi Mizowaki has received honoraria from Varian Medical Systems Inc.; Elekta K.K., Hitachi Ltd., and Brainlab AG played a consulting or advisory role for Varian Medical Systems Inc.; Hitachi Ltd.; has research funding from Hitachi Ltd. and educational projects from Varian Medical Systems and Brainlab AG. Other authors declare no conflicts of interest.

## Data Availability

The data that support the findings of this study are available from the corresponding author upon reasonable request.
